# On the Comparative Periods of Decay of the Bicuspid and Molar Teeth at Different Periods of Life in the Sexes

**Published:** 1854-04

**Authors:** W. A. Pease

**Affiliations:** Dayton, Ohio


*On the comparative periods of decay of the Bicuspid and Molar Teeth at different periods of life in the sexes.* By W. A. Pease, Dayton, Ohio.

We are aware that in preparing this table, we may be regarded by some as performing a work of supererogation—that neither dental or medical science requires the expenditure of so much labor, and that the question will be asked—"cui bonis will it tend?" Of what use is it to collect several hundred ladies and gentlemen together; ask their ages; ascertain their peculiarities of constitution, and examine their mouths, and note the progress of disease in their teeth? It is surely sufficient to know that teeth decay at different periods of life from causes at present beyond our knowledge or control and that treatment is too often but palliation, procrastinating a fixed and inevitable result, ultimate extraction; and that it is sufficient to combat that disease when it appears, with whatever skill we possess. It is no part of our present purpose to elaborate this paper, or attempt to draw conclusions from the limited number of cases, but simply to present our mite to the common treasure of statistics that bear on the course of disease, and its period of incubation in teeth at different periods of life in the sexes. As few persons resort to a dentist till disease has made considerable progress, it is difficult to ascertain this from observation, and another method was employed, which will afford, perhaps, equally reliable and valuable information—by establishing the period when disease has so far progressed as to destroy and break down their structure, and render extraction necessary. The class of individuals selected was composed of those persons who, from poverty, or other causes, allowed their teeth to decay without any dental treatment, and consequently exhibited the natural course of disease; while from ther habitual exercise and non-stimulating diet, their physical and mental health was above average, and the cleanliness of the mouth, from the same cause, balanced the greater care bestowed on the teeth by the higher circles.

The method adopted was as follows:—When a tooth was extracted for an individual who neither required or expected to have any other operation performed, it was recorded in a book kept for the purpose, wherein the age, sex, maxilla, name and part of the tooth where disease commenced, was indicated by characters. There was also recorded, with equal care, the name of the tooth, and all operations performed on it, for all classes, though in a different form; and from a cursory comparison, it is believed the period of attack will nearly coincide among the wealthy and poor, though slightly in advance in the former class. The cause of preponderance of males over females, arises from the greater attention females bestow on their teeth, and their more general resort to dental skill. Moreover, it will be observed, in corroboration of this, that the number of teeth extracted for females under twenty years of age exceeded the number for males more than one per cent.; and two per cent. is doubtless considerably under the actual amount of disease. The period at which the greatest number of teeth was extracted, was between twenty and twenty-five years of age, when the early attacks of disease had had time to accomplish the destruction of the tooth, and the dens sapientiæ had begun to furnish their quota; and but for them the decrease of disease between twenty-five and thirty had been much larger than it appears, as they furnish seven and a half per cent., out of thirty-one per cent., or nearly one-fourth of the amount A

not unimportant fact is disclosed, that the most vulnerable point of the molaris is the suture in the crown; the next is the front side, while the labial side of the third molaris is nearly as obnoxious to disease as the suture in the crown. It may not be without significance, in fixing the value of teeth to which to attach artificial substitutes, to know that between twenty and twenty-five years of age, thirty-two per cent. of what are usually termed double teeth, give out, and at thirty an additional thirty-two per cent. must be added. At twenty-five two per cent. of the first superior bicuspids; four of the second; eight of the first molaris, and three of the second are lost; and at thirty, an additional two per cent. of the first bicuspid; two, and two and a half of the second; six of the first molarsis, and two of the second also yield to disease. These being the teeth to which substitutes are generally attached, the increased ratio of disease, from the wear and uncleanliness of clasps, can be estimated.

Note.—In explanation of the tables appended, which accompany Dr. Pease's article, we would say that the letter C stands for the decay in the crown or center of the tooth; the letter F for front approximal, (locating the decay at that point); the letter P for posterior approximal; and L for decay on the labial face of the tooth.

We have, in further illustration of the tables, say, in the male under fifteen, no decay in the bicuspids at any point, but we have six with decay in the crown or center; three with decay in the anterior approximal face, and one with decay in the labial face. These decays are all on the anterior molar.

The figures at the bottom of each table, we believe, are to designate the number of teeth of each class examined.

It will be well enough, however, to recollect that the tables do not specially refer to the commencement of caries, for this has progressed to that stage which demands the loss of the teeth.

The practice of extracting the anterior molar, to give room for the teeth when crowded, would appear generally to be advisable, for we have in each table a larger proportion of these decayed at an early age—say 15 years.—Ed.

### SUPERIOR MAXILLA—FEMALE.

| | Bicuspid. | | 1st bicus. | | 2d bicus. | | 1st molar. | | | | 2d. molar. | | | | 3d. molar. | | | | |
|---|---|---|---|---|---|---|---|---|---|---|---|---|---|---|---|---|---|---|---|
| AGE. | 1st crown. | 2d. crown. | f. side. | p. side. | f. side. | p. side. | crown. | f. side. | p. side. | l. side. | crown. | f. side. | p. side. | l. side. | crown. | f. side. | p. side. | l. side. | |
| Under 15 | | | | 1 | | 1 | 10 | 1 | | | | | | | | | | | 13 |
| 20 | | | 4 | 2 | 4 | 5 | 19 | 2 | 2 | | 2 | | 1 | 1 | 1 | 1 | | | 44 |
| 25 | | 1 | 7 | 16 | 11 | 10 | 20 | 9 | 2 | 1 | 7 | | 1 | 1 | 8 | | | 4 | 98 |
| 30 | | | 3 | 9 | 14 | 6 | 17 | 7 | 1 | | 9 | | 1 | 1 | 12 | 1 | | 8 | 88 |
| 35 | | | 3 | 4 | 8 | 6 | 4 | 1 | 2 | 1 | 4 | 1 | 1 | | 4 | 1 | | 3 | 43 |
| 40 | | | 1 | | | 1 | 1 | 1 | | | 1 | | | | 3 | | | | 8 |
| 50 | | | | | | | | | | 1 | | | | | | | | 1 | 2 |
| | | 1 | 18 | 32 | 37 | 29 | 71 | 21 | 7 | 3 | 23 | 1 | 4 | 3 | 28 | 3 | | 16 | 297 |
| | | | 50 | | 67 | | 102 | | | | 31 | | | | 47 | | | | |

### INFERIOR MAXILLA—FEMALE.

| AGE. | | | | | | | | | | | | | | | | | | | |
|---|---|---|---|---|---|---|---|---|---|---|---|---|---|---|---|---|---|---|---|
| Under 15 | | | 1 | | | | 17 | 3 | | 1 | 1 | | | | | | | | 23 |
| 20 | | | 1 | | 4 | 3 | 9 | 4 | 1 | 1 | 4 | 2 | 1 | 2 | 3 | | | | 35 |
| 25 | | | 1 | 1 | 2 | 1 | 32 | 8 | 6 | 2 | 20 | 2 | 2 | 1 | 14 | | 1 | 6 | 99 |
| 30 | | 3 | 1 | 1 | 3 | 7 | 24 | 9 | | 2 | 14 | 3 | 1 | 2 | 16 | 2 | | 9 | 97 |
| 35 | | | 3 | 2 | 2 | 1 | 9 | 6 | | | 4 | 1 | | | 11 | | | 3 | 42 |
| 40 | | 1 | 1 | 1 | | | 1 | 1 | 1 | | | | | | | | | | 6 |
| 50 | | | 1 | | | | | | | | | | | | | | | 1 | 2 |
| | | 4 | 9 | 5 | 11 | 12 | 92 | 31 | 8 | 6 | 43 | 8 | 4 | 5 | 44 | 2 | 1 | 19 | 304 |
| | | | | | | | | | | | | | | | | | | | 297 |
| | | | | | | | | | | | | | | | | | | | TOTAL-601 |
| | | | 14 | | 27 | | 137 | | | | 60 | | | | 66 | | | | |
| | 1st bicuspld. | | | | 2d. bicus. | | 1st molar. | | | | 2d molar. | | | | 3d molar. | | | | |

### SUPERIOR MAXILLA—MALE.

| AGE; | 1st bic. | 2d bic. | 1st bic. | | 2d bic. | | 1st molar. | | | | 2d molar. | | | | 3d molar. | | | | |
|---|---|---|---|---|---|---|---|---|---|---|---|---|---|---|---|---|---|---|---|
| | c. | c. | fs | ps | fs | ps | c. | fs | ps | ls | c. | fs | ps | ls | l. | fs | ps | ls. | |
| Under 15 | | | | | | | 6 | 3 | | 1 | | | | | | | | | |
| 20 | | | 5 | | 2 | 2 | 10 | 4 | 1 | 1 | 1 | | 1 | 2 | 1 | | | | |
| 25 | | | 8 | 7 | 7 | 20 | 22 | 19 | 10 | | 10 | 2 | 1 | 2 | 4 | 1 | 1 | 1 | 115 |
| 30 | | | 3 | 13 | 8 | 6 | 31 | 7 | 11 | | 9 | 2 | 1 | | 11 | 7 | | 3 | 112 |
| 35 | 1 | 1 | 8 | 2 | 8 | 4 | 13 | 3 | 1 | | 5 | 3 | | | 4 | 4 | 1 | | 58 |
| 40 | | | 4 | 1 | 3 | 2 | 2 | 1 | 1 | | 2 | 1 | 1 | | 1 | 1 | | | 20 |
| 50 | | | | | | | 1 | | | | | | | | 2 | | 1 | | 4 |
| | 1st bicuspid crown | 2d bicuspid crown | 1st bicuspid, front side | do. Post side | 2d bicuspid, front side | do. Post side | 1st molar crown | front side | Post side | Labial side | 2d molar crown | front side | Post side | Labia side | 3d molar crown | front side | Post side | Labial side | |
| | 1 | 1 | 28 | 23 | 28 | 34 | 85 | 37 | 24 | 2 | 27 | 8 | 4 | 4 | 23 | 13 | 3 | 4 | 349 |
| | | | 52 | | 63 | | 148 | | | | 43 | | | | 43 | | | | |

### INFERIOR MAXILLA—MALE.

| AGE- | | | | | | | | | | | | | | | | | | | |
|---|---|---|---|---|---|---|---|---|---|---|---|---|---|---|---|---|---|---|---|
| Under 15 | | | | | | 2 | 23 | 7 | 1 | | 3 | | | | | | | | 36 |
| 20 | | | | 2 | 1 | 1 | 6 | 8 | 1 | | | 1 | | | 3 | | | | 23 |
| 25 | | | 2 | 4 | 2 | 2 | 31 | 13 | 14 | | 21 | 6 | 2 | 2 | 8 | 4 | | 4 | 115 |
| 30 | | | | | 3 | 7 | 23 | 13 | 8 | 4 | 17 | 4 | 2 | 1 | 19 | 3 | 2 | 6 | 112 |
| 35 | | | | 2 | 3 | 2 | 9 | 5 | 3 | 1 | 11 | 2 | 1 | 2 | 15 | 1 | | 4 | 60 |
| 40 | | 1 | | | 1 | 2 | 4 | 1 | | | 2 | 1 | | | 5 | 1 | 2 | 2 | 29 |
| 50 | | | | | | | 3 | | | | | | | | | | | | 3 |
| | | 1 | 2 | 8 | 10 | 16 | 99 | 47 | 27 | 5 | 54 | 14 | 5 | 5 | 50 | 9 | 4 | 16 | 371 |
| | | | | | | | | | | | | | | | | | | | 349 |
| | | | 10 | | 27 | | 177 | | | | 88 | | | | 79 | | | T | TAL-720 |

